# A visual test based on a freeware software for quantifying and displaying night-vision disturbances: study in subjects after alcohol consumption

**DOI:** 10.1186/1742-4682-11-S1-S1

**Published:** 2014-05-07

**Authors:** José J Castro, Carolina Ortiz, Antonio M Pozo, Rosario G Anera, Margarita Soler

**Affiliations:** 1 Laboratory of Vision Sciences and Applications, Department of Optics, University of Granada, Faculty of Sciences, Avenida de Fuentenueva, s/n 18071 Granada, Spain

## Abstract

**Background:**

In this work, we propose the Halo test, a simple visual test based on a freeware software for quantifying and displaying night-vision disturbances perceived by subjects under different experimental conditions, more precisely studying the influence of the alcohol consumption on visual function.

**Methods:**

In the Halo test, viewed on a monitor, the subject's task consists of detecting luminous peripheral stimuli around a central high-luminance stimulus over a dark background. The test, performed by subjects before and after consuming alcoholic drinks, which deteriorate visual performance, evaluates the influence that alcohol consumption exerts on the visual-discrimination capacity under low illumination conditions. Measurements were made monocularly and binocularly. Pupil size was also measured in both conditions (pre/post). Additionally, we used a double-pass device to measure objectively the optical-quality of the eye and corroborate the results from the Halo test.

**Results:**

We found a significant deterioration of the discrimination capacity after alcohol consumption, indicating that the higher the breath-alcohol content, the greater the deterioration of the visual-discrimination capacity. After alcohol intake, the graphical results showed a greater area of undetected peripheral stimuli around the central high-luminance stimulus. An enlargement of the pupil was also observed and the optical quality of the eye was deteriorated after alcohol consumption.

**Conclusions:**

A greater influence of halos and other night-vision disturbances were reported with the Halo test after alcohol consumption. The Halo freeware software constitutes a positive contribution for evaluating nighttime visual performance in clinical applications, such as reported here, but also in patients after refractive surgery (where halos are present) or for monitoring (time course) some ocular pathologies under pharmacological treatment.

## Background

In recent years, human visual performance has been widely studied using different visual functions such as visual acuity, stereoacuity, or contrast sensitivity function. These visual functions have been tested under different experimental conditions: after ocular refractive surgery, with different illumination levels, and after the intake of different substances, such as alcoholic drinks [1-3]. However, night-vision disturbances mentioned by subjects (such as halos, glare or starbursts) have been studied little, in the most cases using questionnaires, these being a subjective and non-quantitative method of gathering information which is incomplete and imprecise. Only a few works have studied theses disturbances using an objective and quantitative method in subjects having ocular pathologies [4] or after refractive surgery [5], [6]. Regarding other experimental conditions, such as under the effects of alcohol, different works have tested the influence of alcohol consumption on different visual functions, such as the contrast sensitivity or the stereoacuity [7]. However, no studies are available examining this influence on the visual-discrimination capacity under low illumination conditions, a key visual function in daily tasks, such as night-time driving, especially in subjects under the effects of alcohol. In such situations, it becomes necessary to evaluate visual disturbances at night that could diminish visual performance. In this work, we seek to characterize these visual disturbances quantitatively by using a simple visual test that could be applied universally, giving complete information on such disturbances. For this, we used a visual test, the Halo test, based on a freeware software, with no additional hardware, which quantify (by a numerical index) and display (showing the shape), the visual disturbances perceived by the subject under different experimental conditions. We evaluated the visual-discrimination capacity under low illumination conditions before and after an alcohol consumption, an important clinical study, especially in driving. With this freeware software, we provide a simple visual test that could be useful for clinical applications, enabling the evaluation of a key aspect of visual performance under different experimental conditions.

## Methods

The study included a total of 56 subjects (112 eyes) with ages ranging from 20 to 59 years (mean age 26.3 ± 7.7 years, standard deviation included). The admission criteria for the subjects were that all observers had to be moderate social drinkers older than 18 years old, and not being under any pharmacological treatment that could affect their health after alcohol consumption. Furthermore, they had to reach a monocular visual acuity ≥ 1.0 in both eyes (with best optical correction) and no pathological conditions that could affect visual performance, checked by a fundus examination and a biomicroscopy evaluation (using a slit-lamp). All participants in the experiments gave their informed consent in accordance with the Helsinki Declaration. The refraction state for the studied eyes was: 38 emmetropes (no refractive error), 54 myopic and 20 hyperopic, and the corresponding mean refractive error (spherical equivalent) was -2.6 ± 1.9 D and +0.9 ± 0.7 D, respectively. In the experiments, the visual-discrimination capacity was evaluated using a halometer, the Halo test, under two experimental conditions: pre- and post-alcohol consumption. The participants were invited to consume two or more glasses of red wine as the alcoholic drink (without any food), in order to evaluate visual performance under different blood-alcohol rates. The red wine used was *Ribera del Farbes *(*Pago De Almaraes *wineries, S.L. Benalúa de Guadix, Granada, Spain), a young red with 13.5% alcohol content.

### Halometer: Halo test

The halometer used in this study is a simple device based on a software which allows a visual test to be performed on a monitor to detect and quantify night-vision disturbances perceived by the subject, such as halos, glare or starbursts, as shown in the literature [8]. For that, the visual-discrimination capacity was evaluated in dim ambient. This visual test, called *Halo test*, was conducted using the *Halo v1.0 *software (Laboratory of Vision Sciences and Applications, University of Granada, Granada, Spain), a freeware software and free of charge (downloadable from the laboratory webpage: *http://www.ugr.es/~labvisgr/*), as well as an easy and simple tool (no additional hardware needed), which is easily implemented in clinical practice, as recent studies have demonstrated [4,6]. In the test performed in the experiments, the task of the participant consisted of detecting luminous peripheral stimuli around a main high-luminance stimulus (with the monitor background set in the dark state). One of the great advantages of the Halo test is that it presents spatial and temporal parameters that can be controlled by assigning values, performing the visual test under the experimental conditions needed, depending on the group of observers under study.

**Figure 1:**
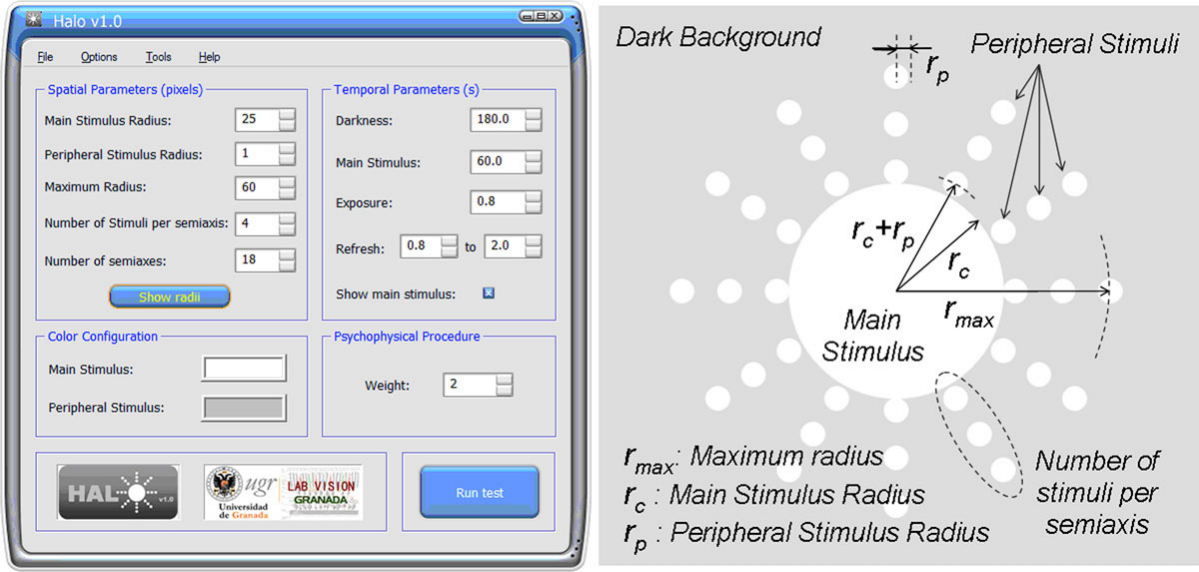
**Halo v1.0 software**. Main window of *Halo v1.0 software *displaying the parameters used in this study (left) and graphic scheme for the spatial parameters of the visual test (right).

Halo v1.0 software has been developed using language C/C++. The user's interface presents a structure similar to that of any other software, as shown in Figure 1, where the main window of Halo v1.0 software can be observed, with different options and information about the configuration of the visual test. Spatial and temporal parameters, as well as the colour configuration and weight, are displayed. All possible parameters are described:

• *Main Stimulus Radius*: the radius, measured in pixels, of the central or main stimulus.

• *Peripheral Stimulus Radius*: the radius, measured in pixels, of the peripheral stimuli presented to the observer around the main stimulus.

• *Maximum Radius*: the distance, in pixels, measured from the centre of the main stimulus to the centre of the farthest peripheral stimulus.

• *Number of Stimuli per semiaxis*: the number of peripheral stimuli distributed in each semiaxis.

• *Number of semiaxes*: the number of semiaxes along which the peripheral stimuli are presented.

• *Show Radii*: this option shows a general graph giving information on the representation of the peripheral stimuli with respect to the semiaxes and main stimulus. It also provides the distance from the centre of the main stimulus to the centre of each of the peripheral stimuli along the semiaxis.

• *Temporal parameters. Darkness*: the time from the start of the test until the central stimulus appears for the first time, i.e. the time dedicated to darkness adaptation (luminance of the monitor background) by the observer.

• *Temporal parameters. Main Stimulus*: the time from the first appearance of the central stimulus until the first appearance of the peripheral stimulus (adaptation time to the main stimulus once the subject is adapted to darkness).

• *Temporal parameters. Exposure*: time that the peripheral stimulus is shown.

• *Temporal parameters. Refresh*: time between peripheral stimuli (from the appearance of a peripheral stimulus to the appearance of the next one). For this time, an upper and lower limit is set by introducing the values in the corresponding boxes. This time is a random value of the interval established, thus minimizing the effect of learning of the subject and avoiding false positives in the detection of the stimuli.

• *Colour configuration*: colour can be configured both for the central stimulus and for the peripheral ones. With this, it is possible to control the luminance of the stimuli.

• *Weight: *the number of times that each stimulus is presented.

Prior to the test, the observer was positioned at 2.5 m from the monitor, using a chin-rest. The luminous stimuli were displayed on a LCD-monitor with the resolution fixed at 1024 × 768 pixels. The test was performed monocularly (left and right eye, contralateral eye occluded) and binocularly, with best correction. The radii of the luminous stimuli were 25 pixels for the central stimulus and 1 pixel for the peripheral stimuli, subtending 0.46 and 0.02 deg, respectively, from observer's position. A 1-pixel radius was used for the peripheral stimulus because participants were emmetropes or ammetropes with best correction and no pathological or diseased eyes. A total of 72 peripheral stimuli around the main one were presented on the monitor, which were distributed along 18 semiaxes (with a ratio of 4 stimuli/semiaxis). The maximum radius of each semiaxis was 60 pixels. For this spatial configuration, peripheral stimuli for each semiaxis were located at 26, 38, 49, and 60 pixels from the centre of the main stimulus.

We used a spectroradiometer SpectraScan PR-650 (PhotoResearch, Inc., Chatsworth, CA, USA) to measure the luminance of the stimuli showed on the monitor. The luminance values were of 175.6 cd/m^2 ^for the main stimulus and of 61.4 cd/m^2 ^for the peripheral stimuli, being the luminance of the monitor dark-state of 0.72 cd/m^2^.

A test session started with an adaption period to the monitor dark-state (3 min) and then an adaptation time to the luminance of the central stimulus (1 min). After this, peripheral stimuli were randomly presented around the central one. The task of the subject was to detect peripheral spots by pressing the left button of the mouse. During the test, observer's answers were stored for later analysis and calculation of the visual disturbance index (VDI). This index is calculated taking into account non-detected stimuli in relation to the total of the peripheral stimuli presented on the monitor (Eq. 1). All terms in the equation are weighted by the squared distance of each peripheral stimulus to the centre of the main one, as well as by the times (over the total weight) that the corresponding peripheral stimulus is not detected by the observer [4].

(1) $$\text{VDI}=\left[{\overset{\text{N}}{\sum }}_{\text{i}=\text{1}}\left({\text{p}}_{\text{i}}\cdot {{\text{r}}_{\text{i}}}^{\text{2}}\right)]/\;[\text{p}\cdot {\overset{\text{N}}{\sum }}_{\text{i}=\text{1}}\left({{\text{r}}_{\text{i}}}^{\text{2}}\right)\right]$$

Where *r_i _*is the distance (in pixels) from the centre of the main stimulus to the centre of the *i*-peripheral stimulus; *N *is the total number of peripheral stimuli; *p *is the total weight (number of times that each stimulus *i *is shown); and *p_i _*is the number of times over the total weight (*p_i _≤ p*) that the *i*-peripheral stimulus is not detected. The VDI varies from 0 to 1. Visual halos around luminous stimuli, glare or other night-vision disturbances referred by the subject will imply a poor discrimination capacity and, therefore, a greater value of the VDI. The VDI's complementary index is the visual discrimination index (VDiscI), which quantifies the visual discrimination capacity by means of the detected peripheral stimuli. This is another way to show the subject difficulties on detecting luminous stimuli around the central light, so that the higher the VDI, the lower the VDiscI. Both indexes are related as follow: VDiscI = 1 - VDI. In this work we used the VDI because is a metric commonly used to characterize visual disturbances perceived by the subject [4,5,8], highlighting in this way the non-detected peripheral stimuli and giving information, together with the graphical results, about the geometry of the visual halo perceived by the subject. In the experiments, we used a weight of 2 (p = 2), which was the same for all the 72 peripheral stimuli shown, taking p_i _values of 0 (peripheral stimulus detected) or 1 or 2 (stimulus not detected once or twice, respectively). Each peripheral stimulus was showed on the monitor for 1s, with a refresh rate ranging from 0.8 to 2s in which only the main stimulus was presented.

As additional useful information, the Halo software provides a graph of results for each test. This graph shows the central stimulus, the non-detected peripheral stimuli and the peripheral stimuli detected. The value showed in the detected stimuli (marked in green colour) indicates the number of times that each peripheral stimulus is detected by the observer over the total weight (1 or 2, for the configuration used in this study), whereas the × (marked in red colour) denote the undetected stimuli. These characters (X, 1, or 2) are placed where the corresponding stimulus was presented. An important aspect of the graph is the shape of the area in which the stimuli were non-detected, providing information about the visual disturbances perceived by the observer, such as visual halos or starbursts, and the areas where visual discrimination capacity is deteriorated.

The experiments were performed under two experimental conditions: pre- and post-alcohol consumption. Both pupil sizes (right and left eye) were measured under the two conditions, after finishing the corresponding test and then performing a new one, using a Colvard pupillometer (OASIS Medical, Inc. Glendora, CA, USA). Additionally, participants performed the test binocularly wearing the alcohol-impairment goggles Alcovista BAC 0.6-0.8º low-level night vision, used in traffic-road-safety education, which simulates night-vision impairment caused by a low blood-alcohol level (estimated breath alcohol content -BrAC- from 0.3 to 0.4 mg/l).

### Breath-alcohol content

To quantify the alcohol content in the body for each subject, we measured the BrAC, in mg of ethanol per litre of exhaled air (mg/l), using the breath analyser *Dräger Alcotest 7110 MK-III *(Dräger Safety AG & Co. KGaA. Lübeck, Germany), which uses two different measuring systems (an infrared sensor and an electrochemical sensor). This instrument is an evidential breath-alcohol analyser used for legal and road traffic purposes in different countries. The device was provided by the traffic police in the province of Granada (*Subsector de Tráfico de la Guardia Civil*. Granada, Spain). Participants were asked to consume the alcoholic drink within a 60-min period. Following this period, the BrAC of each participant was measured three times with the Alcotest breath-analyser: firstly, 30 min after the last drink (just before starting the halo test), secondly, 30 min later, and finally, 90 min after the last drink. For each participant we provided the BrAC as the average of the three measured BrACs.

### Optical-quality measurements

We took objective measurements of the optical quality of the eye in order to show a consistency with the subjective results for the halo test. For that, we used the commercial device OQAS™ (Optical Quality Analysis System, Visiometrics S.L. Tarrasa, Spain) based on the double-pass technique [9,10], which provides data on diffraction, optical aberrations of the eye, and scattering through ocular media that deteriorate the retinal image quality, reducing optical quality of the eye. In this device, a point source (an infrared laser diode, λ = 780 nm) is projected onto the retina. The reflected light passes through ocular media and the double-pass image is recorded by a camera. This objective optical device is useful in patients having an ocular pathology or in patients that have been subjected to some kind of refractive surgery, where part of the ocular media could have been affected or, as in this case, after alcohol consumption, where the tear film is altered [11]. We measured the MTF-cutoff (Modulation Transfer Function cutoff), a parameter commonly used in the optical-quality characterization of an optical system, such as the human eye [9]. The MTF represents the loss of contrast caused by the eye's optics on a sinusoidal grating as a function of its spatial frequency [9]. We analysed the MTF cut-off for a complete evaluation of the optical quality. In theory, this parameter represents the spatial frequency, in cycles per degree (cpd), corresponding to a MTF value of 0.01 (due to the physical noise generated by the CCD camera). A poorer optical quality of the eye results in a lower value of the MTF cut-off. For all participants, a 4-mm pupil size was selected for the measurements with the OQAS.

## Results and discussion

The mean breath-alcohol content for the participants was 0.32 ± 0.14 mg/l, ranging from 0.14 to 0.76 mg/l. Table 1 shows results for objective and subjective measurements: the mean values for the VDI pre- and post-alcohol consumption (monocular and binocularly) and wearing alcohol-simulation goggles (binocularly), as well as the pupil size and the MTF-cutoff (pre- and post-). In both cases, monocular and binocularly, the VDI was significantly higher after alcohol consumption (p < 0.05), indicating a deterioration in the visual-discrimination capacity under low-illumination conditions. Wearing simulation goggles, subjects registered binocular VDI values significantly higher than either binocular VDIs (pre- and post-drinking alcohol), showing an exaggerated effect of the alcohol-consumption simulation on the subject with goggles (all subjects had a VDI higher than 0.85; no one stimulus was detected for some of them). Under both conditions, before and after alcohol consumption, the binocular VDI was significantly lower than the monocular one (p < 0.05). Similar results were found for pupil size, resulting in a significant enlargement of the pupil diameter after alcohol consumption. However, in most cases the pupil-size difference was the sensitivity of the pupillometer (0.5 mm), the difference between the mean pupil sizes (pre/post) being lower than the pupillometer sensitivity.

**Table 1:** Mean values for the disturbance index, pupil size and MTF-cutoff.

	PRE-	POST-	Goggles	p-value
VDI monocular	0.25 ± 0.15	0.39 ± 0.24	-	<0.001
VDI binocular	0.16 ± 0.08	0.26 ± 0.16	0.95 ± 0.05	<0.001
Pupil size (mm)	5.3 ± 0.9	5.6 ± 1.0	-	<0.001
MTF-cutoff (cpd)	40 ± 11	35 ± 12	-	<0.001

Mean values for the visual disturbance index (VDI), monocular and binocular, under different experimental conditions before (pre) and after (post) alcohol consumption and wearing alcohol-impairment goggles (only binocular). Mean values of the pupil size and MTF-cutoff are also showed. Standard deviation included. Statistical analysis: paired-sample t-Test for means.

Regarding the optical-quality of the eye, the MTF-cutoff was significantly lower (p < 0.05) after alcohol consumption, indicating a deterioration of the retinal-image quality under alcohol effect. Before alcohol consumption, the mean MTF-cutoff was of 40 ± 11 cpd (cycles per degree), taking values from 18.9 to 56.3 cpd, whereas the mean MTF-cutoff was of 35 ± 12 cpd after alcohol intake (from 13.1 to 52.4 cpd). The diminishing of the MTF-cutoff after alcohol intake results in a deterioration of the visual function. These results agree with the deterioration of the VDI under alcohol effect, corroborating subjective data from the Halo test with objective data from the double-pass device.

Table 2 shows results before and after alcohol consumption for the visual disturbance index and pupil size according to the BrAC of each subject: participants were classified in two groups attending to the BrAC limit of 0.25 mg/l (established in several European countries for driving purposes): a group with a BrAC≤0.25 mg/l (19 participants, mean BrAC of 0.20 ± 0.03 mg/l) and a high-BrAC group (37 participants, mean BrAC of 0.39 ± 0.13 mg/l), with a BrAC>0.25 mg/. The visual disturbance index, monocular and binocular, was significantly higher after alcohol consumption for both groups (p < 0.001, paired-sample t-Test for means), although differences pre-post were lower for the low-BrAC group (BrAC≤0.25 mg/l). For the pupil size, non-significant differences before and after alcohol intake were found for the low-BrAC group, whereas pupil increased significantly after alcohol intake for the high-BrAC group.

**Table 2:** Mean values for the disturbance index and pupil size for groups with BrAC ≤0.25 mg/l and BrAC > 0.25 mg/l.

	BrAC ≤ 0.25 mg/l		BrAC > 0.25 mg/l	
	**PRE-**	**POST-**	**PRE-**	**POST-**

VDI monocular	0.22 ± 0.10	0.30 ± 0.16	0.27 ± 0.17	0.44 ± 0.25
VDI binocular	0.16 ± 0.08	0.20 ± 0.11	0.16 ± 0.09	0.27 ± 0.17
Pupil size (mm)	5.2 ± 0.9	5.4 ± 1.0	5.3 ± 0.9	5.7 ± 0.9

Mean values for the visual disturbance index (VDI) and for the pupil size before and after alcohol consumption for the two groups: BrAC≤0.25 mg/l and BrAC>0.25 mg/l. Standard deviation included. Statistical analysis: paired-sample t-Test for means.

**Figure 2:**
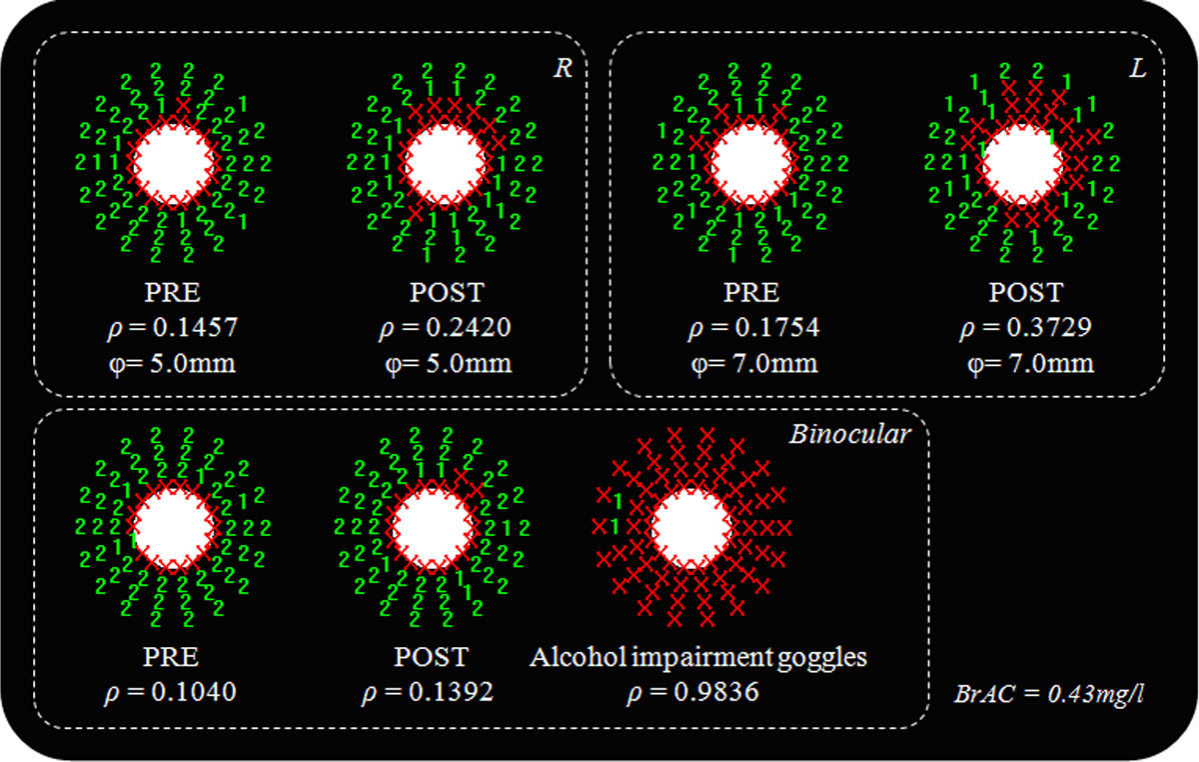
**Graphic results for the Halo test**. Graphic results of the Halo test for a subject with a BrAC of 0.43 mg/l, monocularly (right [R] and left [L] eye) and binocularly, for the different experimental conditions. ρ is the VDI and φ the pupil diameter.

**Figure 3:**
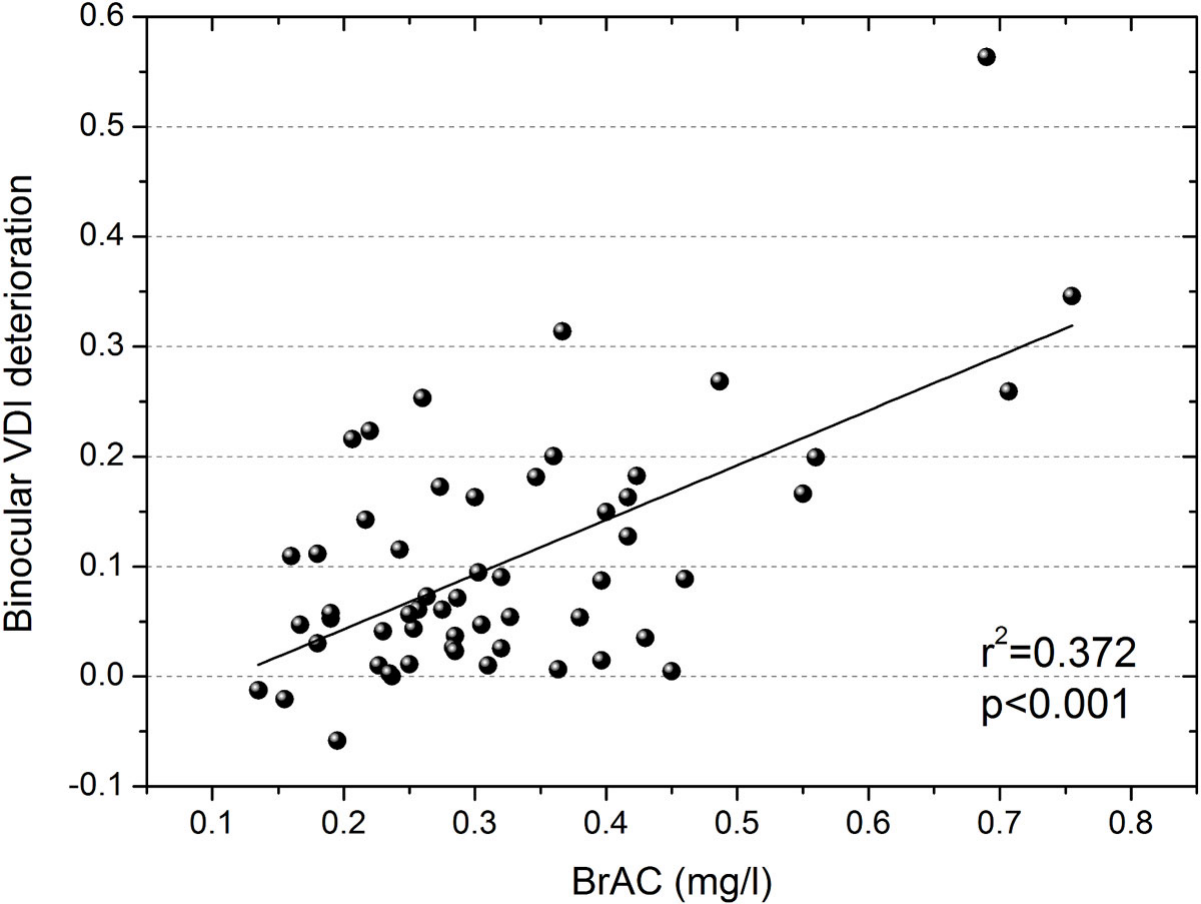
**VDI deterioration Vs. BrAC**. Deterioration for the binocular-disturbance index as a function of the breath alcohol content (BrAC) in mg/l.

Graphical results for the visual test agreed with the numerical values for the VDI. Figure 2 represents the graphical results for a participant under the experimental conditions studied here. Under the influence of halos around the main stimulus, a higher value of the VDI indicates a lower amount of peripheral stimuli detected (X for undetected stimuli; 1 or 2 for detected stimuli once or twice, respectively). The higher the VDI, the higher the halo size around the central luminous stimulus, reducing the visual-discrimination capacity of stimuli close to the main stimulus. Deterioration for the binocular VDI as a function of alcohol content is represented in Figure 3. This deterioration was calculated for each participant as the difference between VDI post- and pre-alcohol consumption, in such as way that a positive value indicates a deterioration in visual-discrimination capacity after having alcoholic drinks. We found a significant ascending correlation (p < 0.001, r^2 ^= 0.372) for VDI deterioration with averaged BrAC (mg/l): the higher the breath-alcohol content, the higher the deterioration for the visual-discrimination capacity.

## Conclusions

In this work, we studied the influence of alcohol consumption on the visual-discrimination capacity under low-illumination conditions. For this, we used a simple visual test, based on a freeware software. Our results showed a deterioration of the visual discrimination capacity after oral alcohol intake, so that the higher the breath-alcohol content, the higher the deterioration of the visual-discrimination capacity, this trend being confirmed when classifying participants in two groups attending to the BrAC limit (0.25 mg/l). This results in a greater influence of halos and other night vision disturbances, which impede peripheral detection of a central visual stimulus, deteriorating visual performance. These results were corroborated with data showing a deterioration in the optical-quality of the eye after alcohol consumption, by means of analyzing the retinal-image quality. With alcohol-impairment goggles, the visual-discrimination capacity is seriously deteriorated, showing exaggerated impairment compared with real situations after alcohol intake. The binocular-discrimination capacity was also significantly higher than the monocular one, showing a positive binocular summation, that is, a positive interaction between the two monocular systems to make a binocular perception of a psychophysical task, increasing visual efficiency. With this, the superiority of the binocular system is demonstrated, in accordance with other studies [12,13]. On average, the pupil diameter was higher after alcohol consumption, increasing optical aberrations of the eye and contributing to perception of visual disturbances such as halos. Furthermore, alcohol consumption disturbs the tear film, as other authors have demonstrated [11], contributing to the reduction in retinal-image quality, as demonstrated with data from the OQAS device, and thus deteriorating visual performance, as reported here. However, in clinical applications, the use of optical instrumentation such as the double-pass device used in this work, is a complicated task due to the high costs of such devices or the space needed in the examination room. Although optical quality of the eye is deteriorated by an increased pupil and an altered tear film, the deterioration in the visual-discrimination capacity could be also affected by changes in the neural performance due to alcohol consumption, altering some tasks, as the visual reaction time or the concentration ability. However, graphical results, as shown in Figure 2, indicate a low impact of subject distraction on the Halo test, due to the halo shapes found in almost all of the graphical results.

Visual tests based on a freeware software, as the Halo test, present great advantages in this sense due to a high simplicity, reliability and availability. As a final conclusion, the use of a universal and a freeware software as a visual test offers a positive and major contribution for evaluating night visual performance in clinical applications, as reported here, but also in patients after refractive surgery (where halos are present) [5,6] or for monitoring (time course) some ocular pathologies under pharmacological treatment [4], showing the power and utility of these types of softwares.

## Competing interests

The authors declare that they have no competing interests.

## Authors' contributions

JJC contributed on the conception and design of the study, acquisition and interpretation of data, drafting the article and approving the version to be published. **CO **carried out the acquisition and interpretation of data, drafting the article and approving the version to be published. **AMP **contributed on the acquisition and interpretation of data, revising the article critically for important intellectual content and approving the version to be published. RGA contributed on the conception of the study, revising the article critically for important intellectual content and approving the version to be published. MS contributed on the acquisition and interpretation of data, and approving the manuscript to be published.

## Declarations

This research was supported by Junta de Andalucía, grant P07-FQM-02663. Publication of this article has been funded by the FQM-151 Research Group.

This article has been published as part of *Theoretical Biology and Medical Modelling *Volume 11 Supplement 1, 2014: Selected articles from the 1st International Work-Conference on Bioinformatics and Biomedical Engineering-IWBBIO 2013. The full contents of the supplement are available online at http://www.tbiomed.com/supplements/11/S1.
